# Impact of Propeptide Cleavage on the Stability and Activity of a Streptococcal Immunomodulatory C5a Peptidase for Biopharmaceutical Development

**DOI:** 10.1021/acs.molpharmaceut.3c00207

**Published:** 2023-07-05

**Authors:** Vinayakumar Gedi, Francisco Duarte, Pratikkumar Patel, Promita Bhattacharjee, Malgorzata Tecza, Kieran McGourty, Sarah P. Hudson

**Affiliations:** † Department of Chemical Sciences, Bernal Institute, University of Limerick, Limerick V94 T9PX, Ireland; ‡ SSPC SFI Research Centre for Pharmaceuticals, University of Limerick, Limerick V94 T9PX, Ireland

**Keywords:** biopharmaceutical, propeptide cleavage, protein stability, C5a peptidase, protein purification

## Abstract

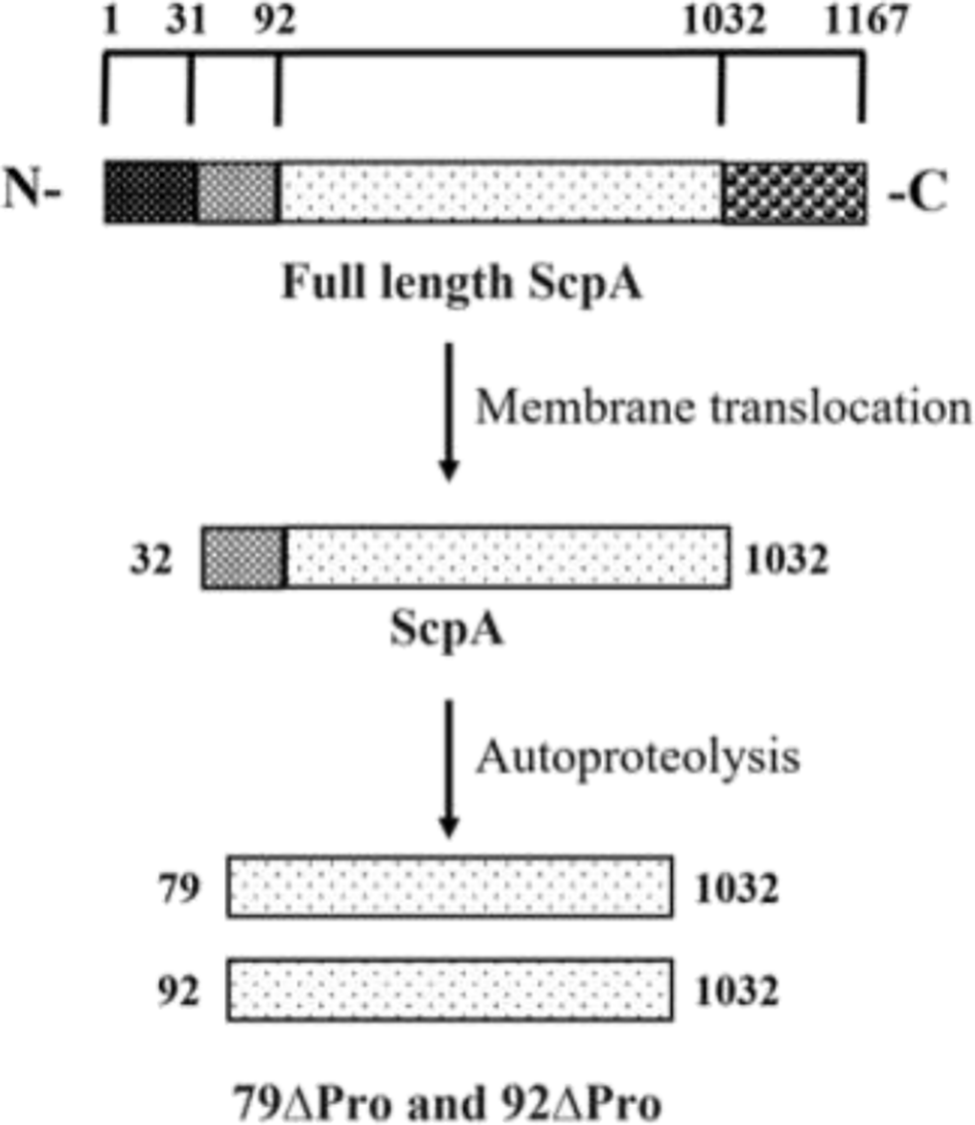

Posttranslational modifications of proteins can impact their therapeutic efficacy, stability, and potential for pharmaceutical development. The Group A*Streptococcus pyogenes*C5a peptidase (ScpA) is a multi-domain protein composed of an N-terminal signal peptide, a catalytic domain (including propeptide), three fibronectin domains, and cell membrane-associated domains. It is one of several proteins produced by Group A*S. pyogenes*known to cleave components of the human complement system. After signal peptide removal, ScpA undergoes autoproteolysis and cleaves its propeptide for full maturation. The exact location and mechanism of the propeptide cleavage, and the impact of this cleavage on stability and activity, are not clearly understood, and the exact primary sequence of the final enzyme is not known. A form of ScpA with no autoproteolysis fragments of propeptide present may be more desirable for pharmaceutical development from a regulatory and a biocompatibility in the body perspective. The current study describes an in-depth structural and functional characterization of propeptide truncated variants of ScpA expressed in*Escherichia coli*cells. All three purified ScpA variants, ScpA, 79ΔPro, and 92ΔPro, starting with N32, D79, and A92 positions, respectively, showed similar activity against C5a, which suggests a propeptide-independent activity profile of ScpA. CE-SDS and MALDI top-down sequencing analyses highlight a time-dependent propeptide autoproteolysis of ScpA at 37 °C with a distinct end point at A92 and/or D93. In comparison, all three variants of ScpA exhibit similar stability, melting temperatures, and secondary structure orientation. In summary, this work not only highlights propeptide localization but also provides a strategy to recombinantly produce a final mature and active form of ScpA without any propeptide-related fragments.

## Introduction

Group A*Streptococcus pyogenes*(GAS) is a Gram-positive pathogenic bacteria accounting for a number of infectious diseases to humans.^1,2^ Its potential virulence factors and their mechanism of virulence have been reviewed elsewhere.^3^ Among others, streptococcal C5a peptidase (ScpA) is a cell-surface-expressed virulence factor in GAS known to play a key role by cleaving complement system factor C5a.^4,5^ C5a is a 74-amino-acid biologically active peptide that plays a crucial role in mediating various immune and inflammatory responses. However, excessive production of C5a is associated with several inflammatory and autoimmune diseases such as adult respiratory distress syndrome, sepsis, psoriasis, multiple sclerosis, and Alzheimer’s disease.^6^

In addition to its virulence characteristic, research on ScpA also focused on its ability to target C5a as an immune modulatory enzyme^7^ and as a potential target for vaccine development.^8−11^ Other recombinant enzymes expressed in bacteria, similar to ScpA, are being explored as potential treatments for many pathologies and conditions. Improvements around monitoring activation of patients’ immune systems as well as the use of encapsulated delivery mechanisms are being developed to mitigate adverse anti-enzyme immune responses.^12,13^ Functionally, ScpA was characterized to be a highly specific protease and cleaves C5a between H67 and K68.^5,14^ The crystal structure of ScpA and its interactions with C5a highlighted key structural features on the activity and ability to cleave C5a.^15,16^ Overall, ScpA is classified as a multi-domain protein with an N-terminal signal peptide, a propeptide, a catalytic domain, three Fn domains, and C-terminal membrane-associated domains.^15^ After membrane translocation, the propeptide can be digested either via autoproteolysis or with the help of external *Streptococcus* cysteine protease streptopain (SpeB) for full maturation.^17,18^

In general, propeptides in proteins play a variety of roles including inhibition of activity in zymogen form and assisting in mature protein folding.^19,20^ Several functions have been determined for the propeptide in serine proteases, including promotion of the correct folding and inhibition of mature protease activity. For example, in subtilisin E, no protease activity was detected when a mature sequence without propeptide was expressed and purified.^21^ It was also identified that a synthetic propeptide was able to guide the refolding of a denatured subtilisin E into an active form, whereas no activity was detected when the refolding was performed in the absence of the propeptide.^22,23^ Furthermore, the propeptide has also been reported to inhibit the activity of subtilisin.^22^ Collectively, the propeptide plays an important role both in guiding the correct folding and in maintaining an inactive orientation before maturation in subtilisin. The role of a propeptide in aiding correct folding has also been observed in other proteases, including streptopain,^24^ α-lytic protease,^25^ and thermolysin.^26^ Even though the mature forms of these proteases are stable, the propeptide was essential to obtaining the final active and stable forms. Given the biological role of proteases in various regulation processes, protease activation and regulation via propeptide have been exploited for therapeutic applications.^11,27^ Posttranslational modifications, including propeptide cleavage, of therapeutic proteins for biopharmaceutical applications can affect their therapeutic efficacy and biocompatibility.^28^ ScpA, like some other members of the subtilisin-like family of serine proteases, contains a propeptide at the N-terminus, which is removed upon maturation. The length of the propeptide has been predicted to be between N32-D78 from the autoprocessed samples stored at either 4 °C or freeze–thawed.^17^ In comparison, the propeptide length reported from external protease SpeB cleavage was until A89, suggesting the possibility of propeptide length beyond D78.^18^ Like in most proteases, the mechanism of propeptide cleavage in ScpA is unknown.

In this study, an in-depth investigation of ScpA propeptide processing at 37 °C was performed using CE-SDS and the possible final point of processing was determined using MALDI top-down sequencing. Furthermore, intact and propeptide truncated forms of ScpA were recombinantly produced and purified using GST-affinity, ion-exchange, and size-exclusion chromatography techniques. Purified ScpA variant stabilities and activities were assessed before and after exposure to heat and multiple freeze–thaw cycles using a fluorescent-based activity assay, melt curves, and far-UV circular dichroism spectroscopy. Based on the findings from these analyses, the potential of truncated forms of ScpA for development as therapeutic biopharmaceuticals is discussed.

## Experimental Section

### Materials

*Escherichia coliDH5*α cells transformed with expression vector pGEX-6P-3 carrying the truncated ScpA genes (ScpA and 79ΔPro) of*S. pyogenesB220* and pProEXHTb carrying the recombinant human C5a (rhC5aC75) and the purified active site mutated version of ScpA (S512A-ScpA) protein were generous gifts from Dr. Jakki Cooney, University of Limerick, Ireland. A Maurice instrument and Maurice CE-SDS Size Application kit reagents (containing two CE-SDS cartridges, reagent vials, 96-well plates, Maurice 1× sample buffer, separation matrix, conditioning solutions 1 and 2, wash solution, running buffer top and bottom) were purchased from ProteinSimple, a Bio-Techne brand (San Jose, California, USA). Mass spectrometry matrices and calibration standards were purchased from Bruker Daltonik GmbH, Germany. All purification columns and chromatography media were purchased from Cytiva, USA. A size exclusion high-performance liquid chromatography (SEC-HPLC) column AdvanceBio SEC 300A 2.7 μm, 7.8 × 300 mm (Part No. PL1180-5301) was procured from Agilent Ireland. SYPRO Orange dye (5000× concentrated solution in DMSO) was purchased from Sigma. All other chemicals and solvents were of analytical grade and were used as received.

### Expression and Purification of ScpA and C5a

**Figure 1:**
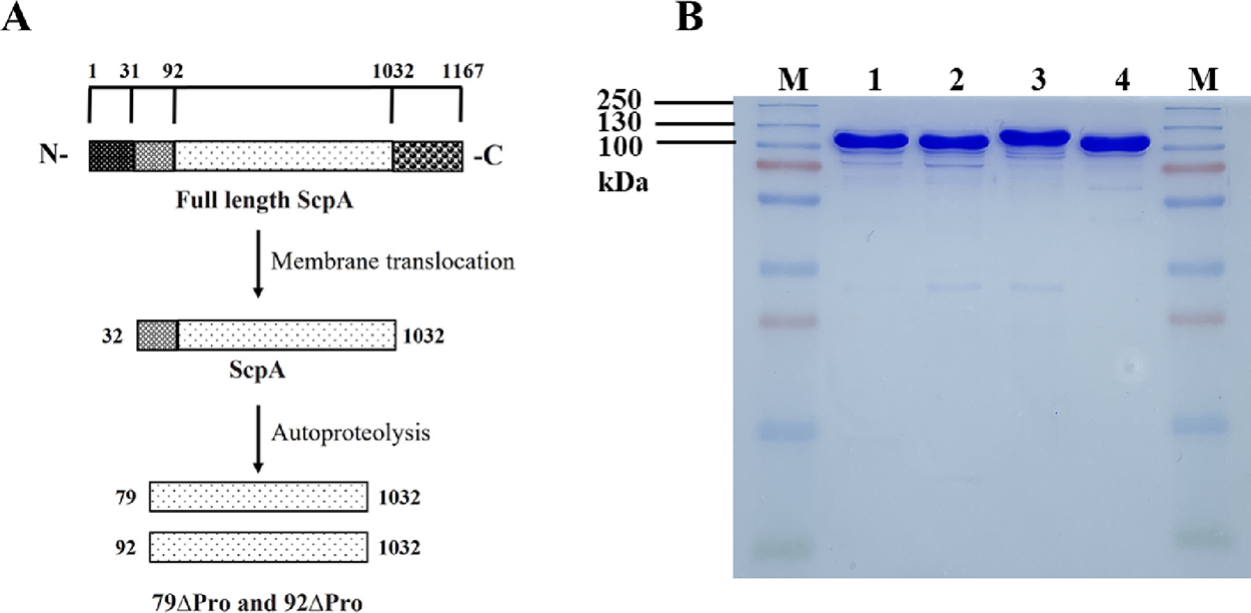
(A) Overall domain organization of ScpA and the variants used in this study. 1–31, signal peptide; 32–91, propeptide; 92–1032, catalytic and Fn1–3 domains; and 1033–1167, cell-wall anchoring domains. (B) SDS-PAGE analysis of purified ScpA variants. (M) Molecular weight ladder, (1) 79ΔPro, (2) 92ΔPro, (3) ScpA, and (4) mature-ScpA.

For easy nomenclature, the variants of ScpA containing amino acids 32–1032, 79–1032, and 92–1032 were called ScpA, 79ΔPro, and 92ΔPro, respectively. Active site serines mutated to alanine versions of 32–1032 and 79–1032 were called S512A-ScpA and S512A-79ΔPro, respectively. The pGEX-6P-3 carrying 92ΔPro and S512A-79ΔPro were generated from site-directed mutagenesis using 79ΔPro as a template. The expression and purification of all four variants of ScpA were carried out as described previously.^15^ Briefly, pGEX-6P-3 carrying the truncated ScpA genes were transformed into*E. coliDH5*α cells, cultured until the absorbance at 600 nm reached 0.6, and induced for 3 h with 0.1 mM IPTG. The cells were further cultured with 25 μg/mL lysozyme for 1 h before harvesting. The harvested pellet from 4 L cell culture was resuspended in phosphate-buffered saline (PBS) containing 1 mg of DNase and incubated for 30 min in cold-room vortexing for lysis. Soluble supernatant containing GST-ScpA was obtained by centrifugation at 18,000 rpm for 30 min and incubated overnight in the cold room with ∼25 mL of GST beads pre-equilibrated in PBS. Note that the GST tag is located at the N-terminus of each ScpA variant. The beads were washed with PBS, and the bound ScpA was then eluted with PreScission protease for 72 h in the cold room. The GST-affinity-purified ScpA was further purified using anion exchange and size-exclusion chromatography, and the concentrated protein aliquots in PBS were stored at −70 °C. PreScission protease-based elution yielded GST-free proteins with more than 90% purity in a single-step high-affinity GST purification (Figure S1). Anion-exchange and size-exclusion chromatography separations were applied to further increase the purity (Figure 1B and Figure S1).

Purified ScpA was used to produce “mature-ScpA” utilizing its ability to autoproteolyse its own propeptide in PBS at 1 mg/mL at 37 °C. The 48 h incubated reaction mixture was then loaded onto GST beads, and size exclusion chromatography was performed to separate the ScpA from the propeptide (mature-ScpA). GST beads utilized here eluted mature ScpA in flow-through by efficiently capturing the cleaved propeptide–GST complex and any remaining uncleaved, immature GST-ScpA. Among the tested time points, ∼90% of propeptide cleavage was observed after 48 h at 37 °C in PBS at 1 mg/mL concentration (data not shown).

The rhC5aC75 with an additional cysteine at the C-terminus (C75 position) was used in this study to assist BODIPY dye quenching. The expression and purification of rhC5aC75 were carried out as described previously.^16^ The BODIPY labeling of rhC5aC75 was also carried out as described previously with slight modifications.^16^ Briefly, 50 μM of rhC5aC75 in 100 mM Tris pH 7.0 and 150 mM NaCl was reduced by the addition of TCEP to a final concentration of 1 mM. The reducing reaction was carried out for 20 min at room temperature in a rotator. The BODIPY dye (final concentration of 2 mM) was then added to the reduced rhC5aC75, and the reaction was continued overnight at room temperature, in a dark environment in a rotator. The PD10 desalting column was used to remove free dye, the fractions (500 μL) were collected, and the protein concentration was determined using A280 nm. The labeling reaction yield was further measured using MALDI-TOF mass spectrometry as described previously.^16^

### Activity Assays

The activity measurements of ScpA variants have been carried out using BODIPY-labeled C5aC75, as reported previously with minor modifications.^16^ Briefly, 200 nM of BODIPY-labeled C5aC75 and indicated concentrations of ScpA in 50 μL of PBS containing 0.1% Tween 20 (PBST) were mixed and scanned for 60 min at 30 s time intervals using a BioTek Synergy H1 fluorescent plate reader with excitation and emission wavelengths of 486 and 525 nm, respectively. The intensities of ScpA-BODIPY-labeled C5aC75 reaction mixtures were subtracted from the BODIPY-labeled C5aC75 alone, and the relative intensities were used for comparative analysis. Unless otherwise mentioned, all the BODIPY-labeled C5aC75 activity assays were performed at ambient temperature.

### ScpA-C5a Binding Assay

Surface plasmon resonance (SPR)-based assay using Biacore X100 was performed to determine the binding affinity between ScpA and C5a, as described previously with slight modifications.^16^ Briefly, active site-mutated forms of both S512A-ScpA and S512A-79ΔPro and histidine-tagged human C5a proteins in HEPES running buffer containing 10 mM HEPES-KOH pH 7.5, 150 mM NaCl, 0.005% (v/v) Tween 20, and 50 μM EDTA were used in this study. The nitrilotriacetic acid (NTA) chip was activated with 0.2 M NiCl_2_ solution (40 s at a flow rate of 10 μL/min) followed by C5a immobilization at a flow rate of 10 μL/min at levels of 25 response units (RU). The binding of both S512A-ScpA and S512A-79ΔPro was performed at various concentrations (5.625, 11.25, 22.5, 45, and 90 nM) at a flow rate of 30 μL/min with association and dissociation times of 180 and 200 s, respectively. All experiments were performed in triplicates.

### Autoproteolysis and Stability of ScpA Variants at 37 °C

The time course of propeptide autoproteolysis of ScpA variants at 37 °C was analyzed after every 24 h for a 1-week period. The analysis was carried out incubating 1 mg/mL of all three ScpA variants in PBS at 37 °C ± 0.5. Aliquots were collected from 0, 24, 48, 72, 96, 120, 144, and 168 h tested for the autoproteolysis end point using CE-SDS and mass spectrometry. The activity of ScpA variants across all time points was also measured for stability analysis. All the experiments were performed in triplicate.

### CE-SDS Analysis

CE-SDS analysis was carried out with the ProteinSimple Maurice system and Maurice CE-SDS Size Application Kit Reagents. The sample preparation for CE-SDS was carried out by diluting ScpA variants to a final concentration of 0.3 mg/mL with the Maurice 1× sample buffer. The reaction mixture of 50 μL containing 15 μL of 1 mg/mL ScpA variants, 1 μL of Maurice CE-SDS 25× Internal Standard (10 kDa recombinant protein), and 34 μL of Maurice 1× sample buffer was loaded into the 96-well plate. Analysis was carried out according to the manufacturer’s instructions. Briefly, the sample 96-well plate and cartridge with top running buffer were inserted into the instrument. Reagents of the kit were placed into the two reagent rows as per the manufacturer’s instructions. The measurement was started by automatic sample injection once for 40 s at 4600 V and separation for 35 min at 5750 V. The detection was performed at a wavelength of 220 nm, and the data was evaluated by Compass for iCE 2.1.0 software.

### Mass Spectrometry Analysis

Intact mass and in-source decay (ISD) analysis of ScpA variants were performed on a Bruker ultrafleXtreme instrument (Bruker Daltonik GmbH, Germany). 2,5-DHAP matrix solution was prepared by mixing a 75:25 volume ratio of 15.2 mg/mL of 2,5-DHAP in ethanol and 18 mg/mL diammonium hydrogen citrate in Milli-Q water. sDHB matrix solution was prepared by dissolving 50 mg of sDHB in 1 mL of 50:50 volume ratio of acetonitrile and 0.1% v/v TFA solution. All protein samples were buffer exchanged with 0.1% v/v TFA solution. For intact mass, 1 μL of ScpA sample (from 1 mg/mL stocks), 1 μL of 2% TFA, and 1 μL of 2,5-DHAP matrix solution were mixed with a pipette until crystallization started and 1 μL of crystalline suspension was deposited onto MALDI ground steel target plates and allowed to dry at room temperature. Spectra were recorded in the linear positive mode, and masses were calculated using externally calibrated Protein Calibration Standard I (Bruker Daltonik GmbH, Germany). For MALDI-ISD, 1 μL of ScpA sample (from 2 mg/mL stocks) and 1 μL of sDHB matrix solution were mixed with a pipette until crystallization started and 1 μL of the crystalline suspension was deposited onto MALDI polished steel target plates and allowed to dry at room temperature. ISD spectra were recorded in the positive reflector mode using the externally calibrated myoglobin standard. All data was acquired and analyzed using the Compass 1.4 software suite from Bruker.

### Circular Dichroism Spectroscopy Analysis

The secondary structural profile of ScpA variants was compared by measuring far-UV CD spectra using the Applied Photophysics Chirascan CD system. The concentrated protein samples were buffer exchanged via dialysis into 10 mM potassium phosphate buffer pH 7.4. For measurements, proteins were diluted to a final concentration of 20 μg/mL in 1 mM potassium phosphate buffer pH 7.4. The spectra were recorded using Pro-Data Chirascan from 190 to 260 nm with a step size of 1 nm, time per point to 1 s, and three repeats. The final spectra were obtained using Pro-Data viewer by taking an average of three repeats and subtracting from baseline.

### Thermal Stability Assays

The temperature effect on ScpA activity was analyzed by incubating ScpA variants (1 mg/mL in PBS) at various temperatures for 30 min followed by cooling on ice. An activity assay was performed at ambient temperature using cooled samples and BODIPY-labeled C5aC75 as described in the activity assay section above. The activity of fresh ScpA from the freezer was considered as control and compared with incubated samples at various temperatures to determine the effect of temperature.

A thermal shift melt curve assay was performed with SYPRO Orange dye (5000× in DMSO) in a 96-well plate using a QuantStudio RT-PCR instrument. The ScpA and dye concentrations used in this assay were 0.2 mg/mL and 5×, respectively. 20 μL of the protein–dye reaction mixture was added to a 96-well RT-PCR plate, and melt curves were measured by holding the plate at 25 °C for 2 min and increasing the temperature from 25 to 99 °C at 0.03 °C/s followed by 2 min at 99 °C. The melt curves were used to calculate Tm values using the TSA-CRAFT online web-based tool.^29^

### Freeze–Thaw Assay

The stability of ScpA and 79ΔPro variants was also tested under repeated freeze–thaw conditions. ScpA (500 μL of 1 mg/mL) in PBS was frozen at −80 °C overnight and thawed the next day for 1 h at 25 °C. The cycle was repeated for 10 times, and the stability of the 10th round freeze–thaw samples was analyzed using activity and SEC-HPLC assays.

### HPLC Analysis

An SEC-HPLC method was developed to analyze the stability and homogeneity of ScpA variants using an Agilent 1200 Infinity Series (Agilent Technologies, USA) and comprised a quaternary pump (G1311B 1260), an ALS autosampler (G1329B 1260), a thermostated column compartment (G1316A 1260), and an MWD VL diode-array detector (G1365D 1260). The chromatographic separations were carried out using an AdvanceBio SEC 300A 2.7 μm 7.8 × 300 mm column. The column was washed with water and equilibrated with the mobile phase (150 mM sodium phosphate, pH 7.0) for 2 h with a flow rate of 1 mL/min. The ScpA samples (5 μL of a 1 mg/mL solution) were injected, and the eluate was analyzed for 15 min, using both 280 and 220 nm detection wavelengths. The peak shape and retention times were analyzed and used for the interpretation of purity. All the measurements were performed in triplicates and the average spectra used for analysis.

## Results

### Production, Purification, and Activity of ScpA Variants with Varying Lengths of Propeptide

Three different variants of ScpA with and without propeptide containing amino acids 32–1032, 79–1032, and 92–1032 were called ScpA, 79ΔPro, and 92ΔPro, respectively (Figure 1).

All variants were purified using similar methodologies, and the purification yield was estimated to be ∼23.2–25 mg/L (Table 1). The molecular weights of ScpA, 79ΔPro, and 92ΔPro calculated from MALDI intact mass analysis were in agreement with the calculated mass from their amino acid sequences (Figure S2, Table 1). In addition, purified ScpA was used to produce “mature ScpA” utilizing its ability to autoproteolyse its own propeptide, as described in the methods section. The migration difference in SDS-PAGE analysis between mature-ScpA and ScpA clearly suggests the propeptide separation (lanes 3 and 4 in Figure 1). Lastly, an inactive 79ΔPro variant where the active site serine was mutated to alanine (S512A-79ΔPro) was also purified for comparison purposes. Thus, five forms of the C5a peptidase were produced and used throughout this study: (i) ScpA, (ii) mature ScpA, (iii) 79ΔPro, (iv) 92ΔPro, and (v) the inactive form, S512A-79ΔPro (Figure 1A).

**Table 1:** Summary of Purification Yield and Molecular Weight Details of ScpA Variants

	amino acid sequence composition	MW calculated from sequence (kDa)	MW calculated from intact mass (kDa)	yield/liter (mg)
ScpA	32–1032	110.310	110.083	25.0
79ΔPro	79–1032	105.343	105.357	24.3
92ΔPro	92–1032	104.050	104.055	23.2

**Figure 2:**
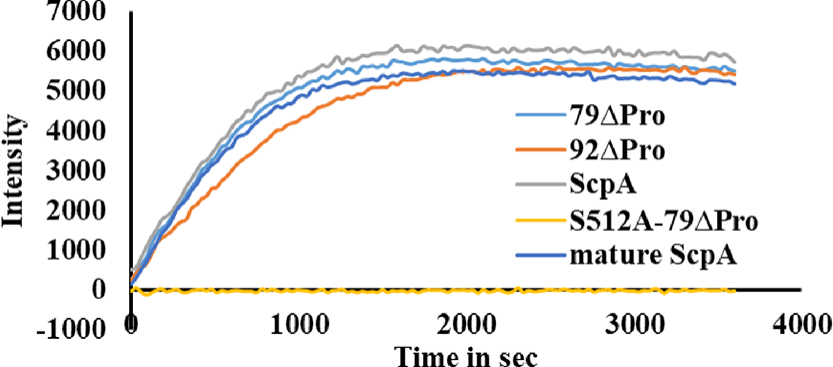
Time-dependent activity of purified ScpA variants (500 pM) tested against BODIPY-FL-C75 (200 nM) in PBS-T.

The activity of purified ScpA variants was analyzed using BODIPY-labeled C5aC75 in a 96-well plate-based fluorescence assay.^16^ As shown in Figure 2, all three purified variants of ScpA and mature-ScpA exhibit similar activity profiles under tested conditions whereas no activity is observed with the mutated S512A-79ΔPro.

### Impact of Propeptide on ScpA Ability to Bind C5a

In addition to the activity assay, the binding affinity between C5a and both ScpA and 79ΔPro were analyzed using SPR. An SPR-based binding affinity study was reported to be highly sensitive where a low nM affinity range was reported for ScpA and C5a.^16^ Like in this previous study, to minimize the complex catalytic feature of active ScpA against C5a, inactive forms of both versions (S512A-ScpA and S512A-79ΔPro) were utilized in this assay.

**Figure 3:**
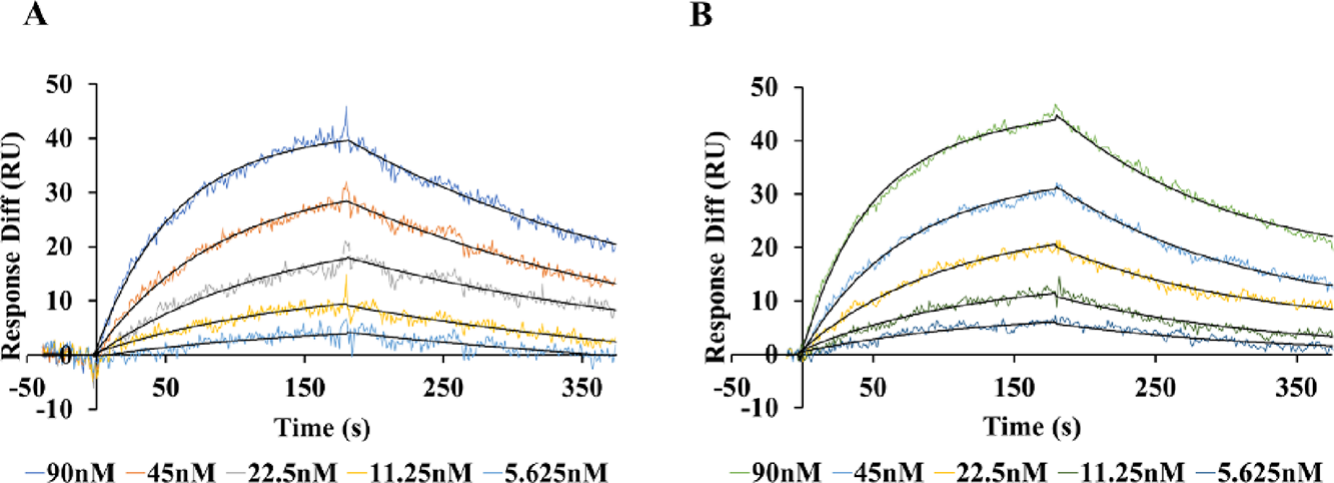
SPR sensograms of both S512A-79ΔPro (A) and S512A-ScpA (B) binding to His-C5a.

As shown in Figure 3, both S512A-ScpA and S512A-79ΔPro sensograms were similar from global fitting of data with a Langmuir model of binding kinetics. The equilibrium dissociation constants (*K*_D_) derived from the represented sensograms were 35 and 25 nM for S512A-ScpA and S512A-79ΔPro, respectively. The *K*_D_ value of S512A-ScpA against C5a determined in this study was in agreement with a previously reported study, 34 nM.^16^ Comparison of other kinetic values between both variants in detail is listed in Table S1. The similar binding affinities of C5a against both ScpA and 79ΔPro further suggest that folding of ScpA and binding to C5a is independent of the presence of the propeptide.

### Autoproteolysis of ScpA

**Figure 4:**
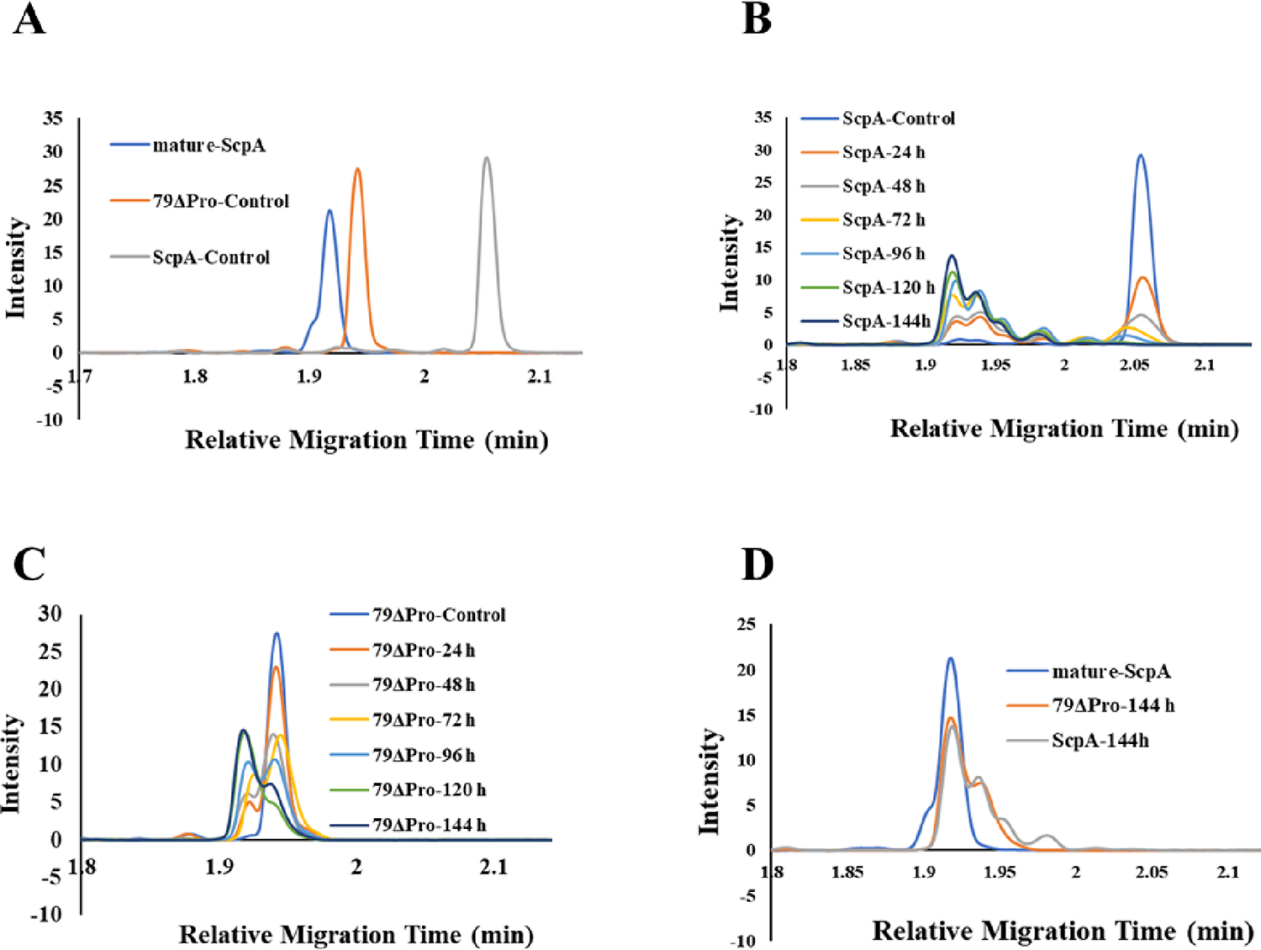
CE-SDS analysis of control and processed ScpA variants. CE-SDS spectra of (A) purified ScpA variants freshly used from freezer, (B) ScpA incubated at 37 °C for 144 h, (C) 79ΔPro incubated at 37 °C for 144 h, and (D) mature-ScpA with 79ΔPro and ScpA after incubation at 37 °C for 144 h.

The current study further focused on identifying the end point of the propeptide position by extensive incubation of the ScpA variants in PBS at biological temperature, 37 °C. The CE-SDS method was selected for its sensitivity to distinguish all three variants of ScpA tested in this study (Figure 4A). Initially, the ScpA variant (with propeptide) was selected and incubated at 37 °C for 144 h, and the extent of propeptide cleavage over time was analyzed (Figure 4B).

As observed in Figure 4B, the main peak of intact ScpA when stored in the freezer decreased and time-dependently shifted when the protease was incubated at 37 °C. More than 90% of ScpA processed the propeptide after 48 h at 37 °C (Figure 4B). The intermediate peaks between the initial and final relative migration times (RMT) suggest the sequential processing of propeptide rather than a one-step cleavage. Furthermore, the RMT of the initial 79ΔPro also shifted and saturated at mature-ScpA RMT when incubated at 37 °C for 144 h (Figure 4C and Table 2). In comparison, the 79ΔPro processing is slower than ScpA, where only ∼50% maturation is observed after 48 h. The slow maturation process may be due to the short propeptide sequence available (N-term starts with D79) for processing when compared with ScpA (N-term starts with N39). The shift in 144 h incubated 79ΔPro clearly suggests that the final point of maturation is further than the reported D79. Finally, the RMT of both 79ΔPro and ScpA final processed peaks were compared with purified mature-ScpA. Notably, the RMT of mature-ScpA and 37 °C processed forms of 79ΔPro and ScpA were comparable (Figure 4D, Table 2).

**Table 2:** Relative Migration Times of Control and 144 h Incubated ScpA Samples at 37 °C

RMT	79ΔPro (min)	ScpA (min)	mature ScpA (min)	92ΔPro (min)
control in freezer	1.942	2.054	1.918	1.915
144 h at 37 °C	1.918	1.919		1.915

**Figure 5:**
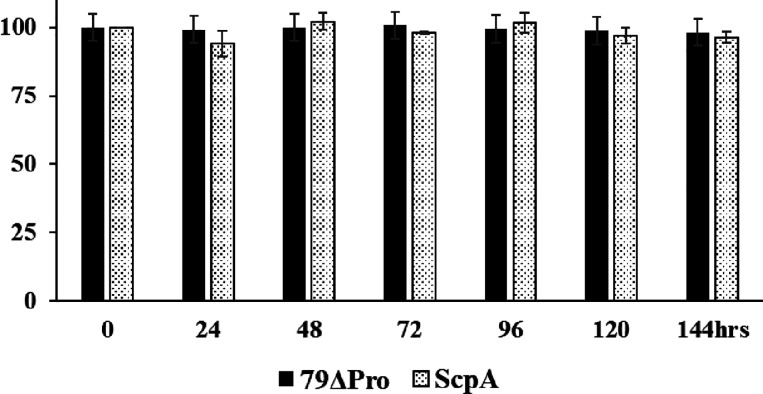
Relative intensities of activities of both 79ΔPro and ScpA incubated at 37 °C. Purified proteins in PBS incubated at 37 °C for indicated time and relative intensities were calculated considering the activity of control protein intensity as 100%.

In addition to the propeptide processing, the effect of incubation at 37 °C on ScpA activity was also tested in this study. Unless otherwise mentioned, the ScpA samples were pre-incubated at indicated periods and the activity assays were performed at room temperature. As shown, more than ∼95% activity of both ScpA and 79ΔPro was retained after incubation for 144 h at 37 °C (Figure 5).

The observation of propeptide processing further than the reported D79 led to an investigation into the sequence of mature ScpA and both ScpA and 79ΔPro. MALDI top-down sequencing was used to sequence both N- and C-terminals of mature ScpA and 37 °C incubated ScpA and 79ΔPro. N-Terminal sequencing of mature ScpA produced in this study revealed three different processed forms of enzyme starting with amino acids K90, A92, and D93 (Figure S3). The sequencing of ScpA incubated at 37 °C for 144 h also revealed high-intensity peaks of a processed form starting with K90 (Figure S4). Similarly, N-terminal sequencing of 144 h incubated 79ΔPro at 37 °C also resulted in three different processed forms starting with K90, A92, and D93 (Figure S5). As expected, no intact forms of both ScpA and 79ΔPro were detected after incubating at 37 °C, suggesting 100% propeptide processing. In comparison, the MALDI-ISD spectra indicate the highest abundance of K90 and A92 forms of ScpA in both variants of ScpA. The invention of 92ΔPro was observed from the sequencing study alone, and thus cloning and purification were performed thereafter. To further confirm the autoproteolysis end point of the different variants, 92ΔPro was also incubated at 37 °C for 144 h and the processing was measured using CE-SDS and top-down sequencing. No shift in RMT was observed in CE-SDS between control and processed forms of 92ΔPro at 37 °C for 144 h (Figure S6, Table 2). In comparison, the CE-SDS peak of 92ΔPro before and after processing at 37 °C exhibits a similar peak profile with mature ScpA (Figure S6B). As expected, N-terminal sequencing showed that predominantly ScpA started with intact (A92) and small quantities of starting with D93 (Figure S7). Intact A92 and D93 versions even after 144 h strongly suggest the potential end point of propeptide processing at A92/D93. No change in the C-terminal sequence was observed in all forms tested in this study (data not shown).

### Impact of Propeptide on ScpA Secondary Structure

**Figure 6:**
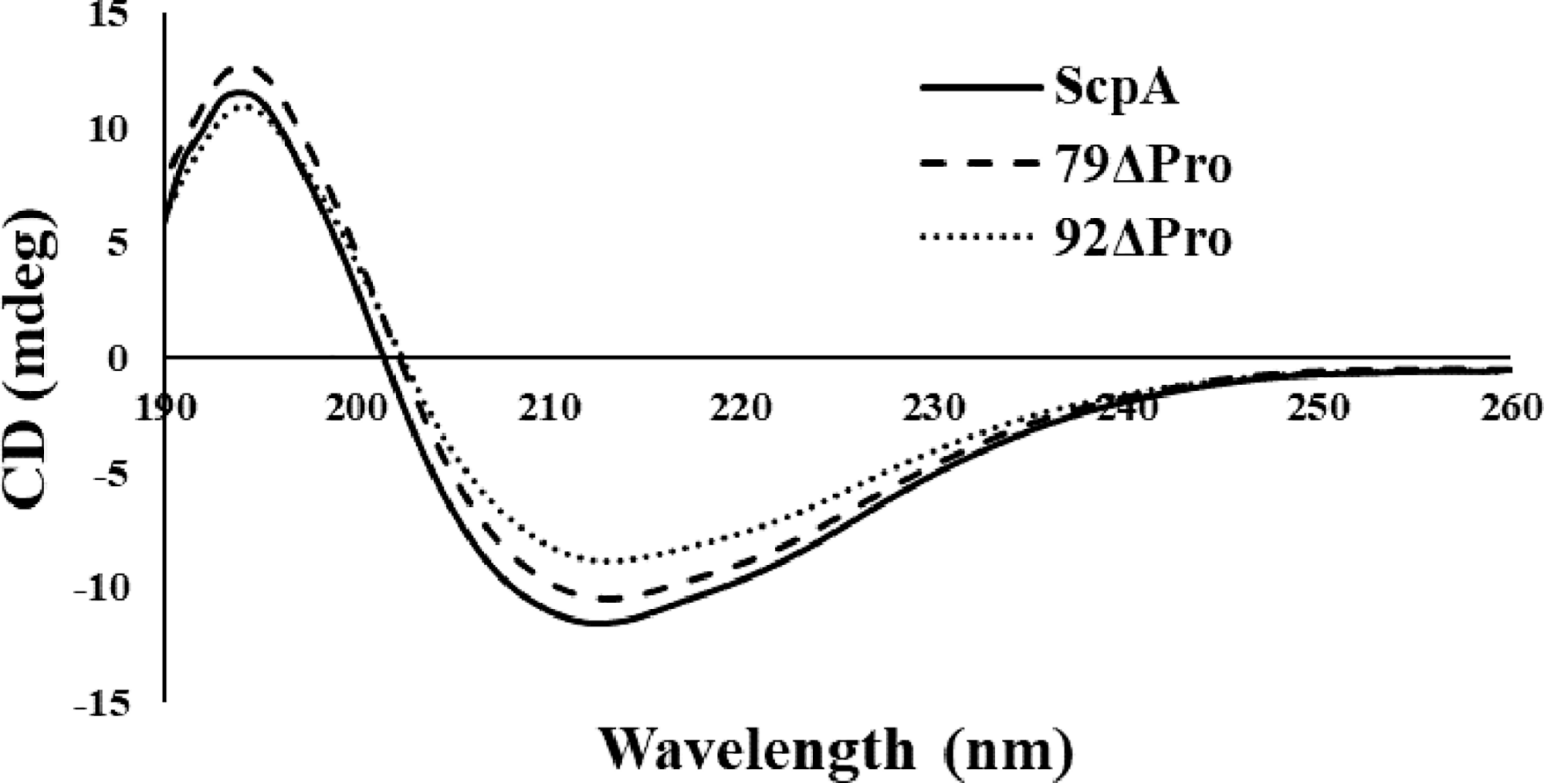
Far-UV CD spectra of ScpA variants.

In the published literature, many techniques have been successfully used to observe structural changes in proteins.^30^ Among these, far-UV circular dichroism (CD) spectroscopy is a simple and excellent method to determine and distinguish any significant secondary structural changes in proteins.^31^ In the current study, far-UV CD spectra were recorded to elucidate differences in ScpA variants expressed and purified with and without the propeptide. All three variants exhibited a similar characteristic positive ellipticity maxima at 194 nm and negative minima between 210 and 215 nm (Figure 6). Similar spectra have been reported for ScpA previously.^17^ Far-UV CD spectra of all three variants suggest no significant changes in the overall secondary structure of ScpA when expressed and purified with and without the propeptide.

### Impact of Propeptide on ScpA Stability

**Figure 7:**
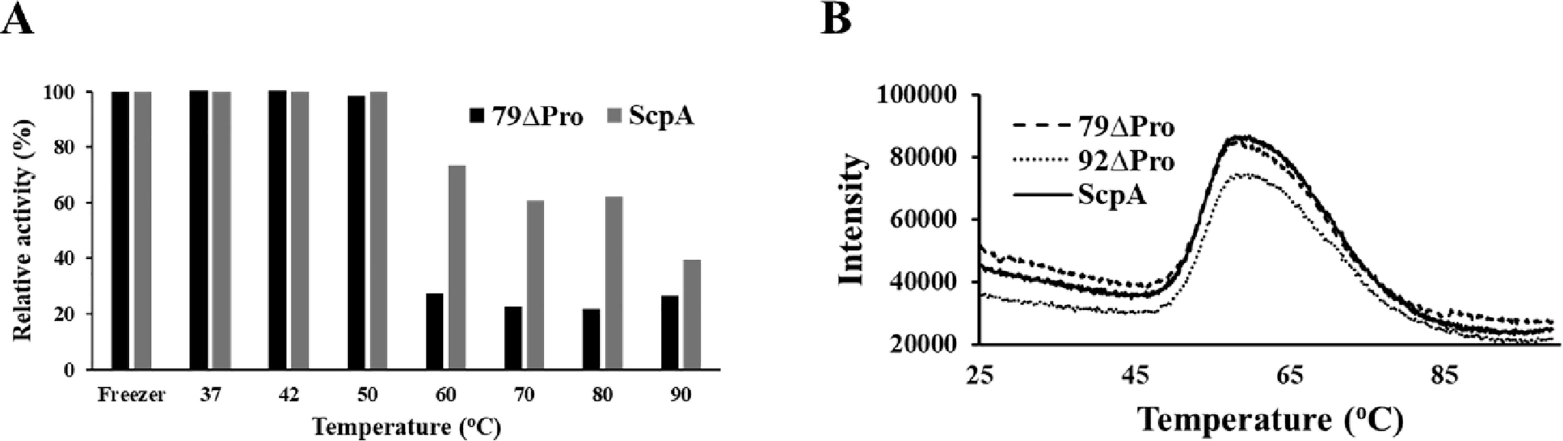
(A) Relative activities of ScpA and 79ΔPro incubated at various temperatures. ScpA variants were incubated at indicated temperatures and cooled on ice, and activity assays were performed at room temperature. (B) Melt curves of all three ScpA variants, ScpA, 79ΔPro, and 92ΔPro, from real-time PCR.

The impact of temperature on the activity of ScpA variants (ScpA and 79ΔPro) was tested by incubating 1 mg/mL solutions of each variant at various temperatures for 30 min and subsequently cooling on ice. As shown in Figure 7A, no activity change was observed until 50 °C. More than 80% activity of 79ΔPro was lost at temperatures above 60 °C. However, ∼60 and ∼40% activity were still observed with ScpA samples incubated at 60–80 and 90 °C samples, respectively. It is worth mentioning that samples were incubated at the indicated temperatures, but the activity assay was performed at room temperature under standard assay conditions. Thus, the activity recovery observed in ScpA may be largely due to its refolding ability when cooled on ice. The activity assay was not performed at pre-incubated temperatures due to limitations of the BODIPY-labeled C5aC75 assay setup.

Furthermore, the role of propeptide in ScpA stabilization was also tested in terms of melting temperature (*T*_m_) using a thermal shift assay, also referred to as differential scanning fluorescence. The thermal shift assay is a rapid and high-throughput method, which is routinely used to measure protein unfolding by increasing the temperature in the presence of a fluorescent dye.^32,33^ With increasing temperature, proteins unfold and expose their hydrophobic cores and then the fluorescent dye can bind, resulting in an increase in fluorescent intensity. Stability can be determined from the temperature-dependent unfolding and *T*_m_ is measured from the midpoint of protein unfolding. Within the tested conditions, all three variants showed similar melt curve patterns (Figure 7B). The intensity starts increasing from 48 °C and reached maximum at 57–60 °C. The *T*_m_ values calculated from the melting curves of all three variants were around 52 °C (Table 3). The similar melt curve pattern and *T*_m_ values of ScpA and its truncated variants further suggested the possible utilization of recombinantly expressed and purified ScpA without its propeptide.

**Table 3:** *T*_m_ Values of ScpA Variants Determined Using Melt Curves from the Thermal Shift Assay

	79ΔPro	ScpA	92ΔPro
*T*_m_ (°C)	52.8	53.2	52.9
*R*^2^	0.99	0.99	0.99

**Figure 8:**
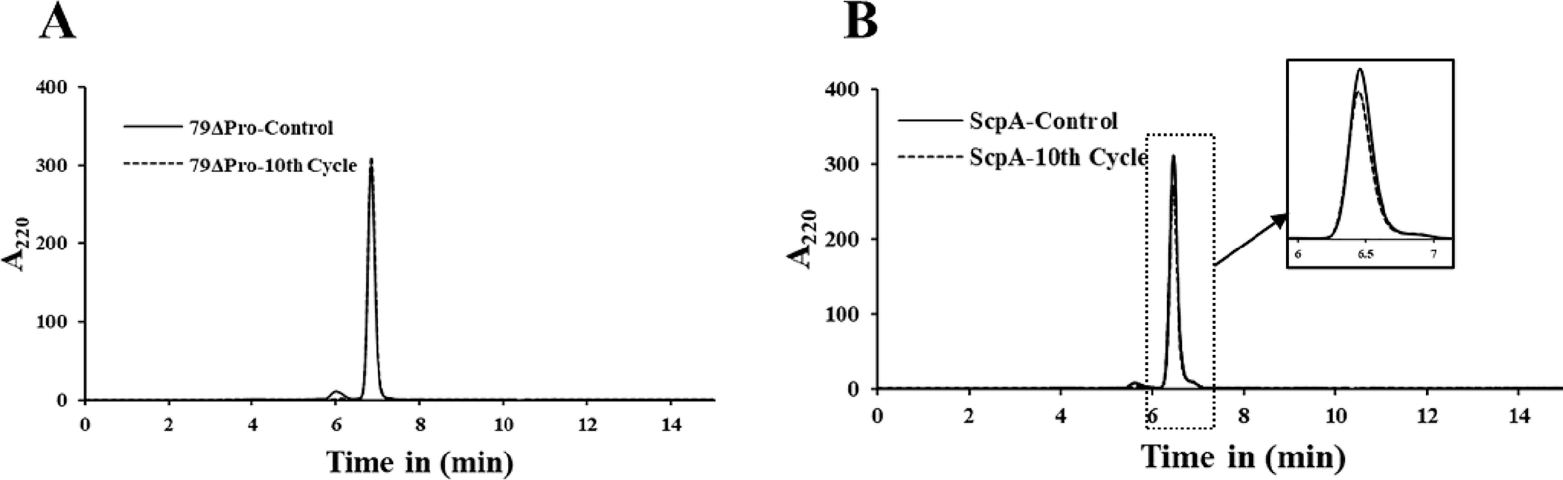
SEC-HPLC chromatogram of freeze–thaw subjected ScpA variants. (A) Initial and 10th-cycle 79ΔPro. (B) Initial and 10th-cycle ScpA (zoomed chromatogram of marked elution time shown in the inset).

Storage at subzero temperatures is an important criterion to increase the long-term shelf life of proteins. The process however requires freezing and thawing, which can sometimes significantly impact a protein’s stability. In this study, both ScpA and 79ΔPro variants were subjected to 10 freeze–thaw cycles and the stability was measured using SEC-HPLC and the described fluorescence activity assay. SEC-HPLC was used here to monitor the purity and also to quantify any soluble aggregates that may have developed during the freeze–thaw cycles. As shown in Figure 8A, no significant change in peak position was detected between the control and 10th-cycle 79ΔPro. However, a slight decrease in peak intensity was observed with the 10th cycle of ScpA in comparison with control ScpA (Figure 8B). The change/decrease in peak intensity may be due to the auto-processing ability of the propeptide. This conclusion is further supported from activity assays where no significant differences in activity were observed between control and freeze–thaw samples of both ScpA and 79ΔPro (Figure S8). The SEC-HPLC results together with the activity assay strongly suggests that the overall stability of ScpA is maintained under repeated freeze–thaw cycles.

## Discussion

Proteases with high substrate specificity and catalytic efficiency are attractive therapeutic candidates with various clinical applications. Rationally engineering the optimal form of proteases, and potentially using gene therapy to ensure sustained delivery of these engineered proteases, could expand the future potential clinical applications for proteases extensively. However, posttranslational modifications, such a propeptide cleavage, could impact the stability, activity, and feasibility of developing such proteases as biopharmaceuticals. ScpA, a cell surface protein in GAS, has been reported to play a key role in cleaving C5a, a component of the complement system.^4,5^ The cleaved C5a has been shown to have a reduced ability to stimulate the leukocyte functions of chemotaxis and exocytosis and thus has attracted interest in ScpA as a therapeutic candidate. ScpA, classified as a member of the subtilisin family of serine proteases, has significant amino acid sequence similarities to subtilisins in regions crucial for enzyme activity.^34^ However, in contrast to subtilisins, ScpA shows high substrate specificity where human complement factors C5a, C3, and C3a are its only reported substrates.^5,35^

ScpA, like other subtilases, is expressed as a multidomain protein and sequentially matures to an active enzyme after membrane translocation and autoproteolysis (Figure 1A). The sequence comparison and previous studies reported the signal peptide and propeptide positions to 1–31 and 32–78, respectively.^17,34^ To characterize the propeptide role, different versions of ScpA with and without propeptide were recombinantly produced to high purity. In comparison, the expression and purification profiles of ScpA, 79ΔPro, and 92ΔPro exhibit similar expression and purification profiles under tested conditions (Figure 1B and Table 1). The SPR-based binding studies also revealed similar binding affinities of both ScpA and 79ΔPro variants against C5a (Figure 3). In addition, the activity profile of mature-ScpA produced by separating propeptide after autoproteolysis of ScpA is also in agreement with that of ScpA, 79ΔPro, and 92ΔPro versions (Figure 2). These results suggest a propeptide-independent folding and activity profile of ScpA in contrast to some other subtilisin family of serine proteases where the propeptide plays a key role in folding and inhibition.^21,23^

The propeptide processing of ScpA has been reported either via autoproteolysis or by another protease, SpeB.^17,18^ The propeptide cleavage is largely believed to be sequential and random. In agreement, Anderson et al. previously reported end points of propeptide processing at A72 of freshly purified and D79 of samples stored at 4 °C and after freeze–thaw cycles of ScpA.^17^ The 79ΔPro selection in this study was based on the aforementioned observation.^17^ To determine the propeptide final processing point, both the ScpA and 79ΔPro versions were incubated at 37 °C and the progress of autoproteolysis followed. MALDI-TOF sequencing of auto-processed forms of ScpA and 79ΔPro identified that matured enzymes started at K90, A92, and D93 whereas sequencing of auto-processed forms of 92ΔPro identified that matured enzymes start with A92 and D93. K90 and A92-starting enzyme peaks were predominantly observed over the D93-starting version. This observation was not a total surprise considering that the SpeB-processed form of ScpA results in a K90 form of ScpA.^18^ These results clearly suggested a time-dependent and sequential rather than single-point processing of the propeptide with the processing end point lying between K90 and D93.

In terms of structural stability, all three purified versions (ScpA, 79ΔPro, and 92ΔPro) showed similar Far-UV CD spectrums and melting temperatures (Figures 6 and 7). Both ScpA and 79ΔPro also showed exceptional stability until temperatures of 50 °C and for up to 10 freeze–thaw cycles (Figures 7A and 8). These results together suggest that propeptide and propeptide truncated expressed versions of ScpA have similar functional activity and stability under tested conditions. Thus, ScpA is active when produced both with and without its propeptide. The propeptide is not required for correct folding like in other bacterial proteases.^21−26^ The propeptide-free production of ScpA results in an active and stable form of the protease comparable to the stable mature form (produced with the propeptide and allowed to autocleave to reach the same active endpoint). However, unlike in other subtilisin families of serine proteases, the exact role of the propeptide and mechanism of autoproteolysis in ScpA is still unknown and warrants further investigation.

## Conclusions

ScpA is considered as a key virulent factor of GAS that cleaves complement component C5a. While the structural orientation and functional characterization of ScpA has been previously reported,^15−17^ the work presented here successfully determined the final end point of propeptide processing as D93. The production and characterization of truncated versions of ScpA also revealed propeptide independent folding and activity in ScpA unlike in other families of subtilisin-based serine proteases. The exceptional stability of ScpA at temperatures up to 50 °C and after multiple freeze–thaw cycles would be advantageous to overcome sensitive stability challenges in production, formulation, and transport and storage processes. While further studies to explore the biological impact of the ScpA maturation process are required, the results in the current study suggest that active, stable, and propeptide truncated versions of ScpA are exciting potential candidates for further research and pharmaceutical applications.

## Supporting Information Available

SDS-PAGE analysis of purification stages of ScpA variants; intact mass spectra of purified ScpA variants; MALDI-ISD spectra of mature-ScpA, ScpA, and 79ΔPro; CE-SDS analysis of 92ΔPro, activity profiles of control and freeze–thaw-subjected 79ΔPro and ScpA variants; and SPR kinetic parameters of both S512A-79ΔPro and S512A-ScpA variants against C5a (PDF)
