# Supplementary material for: Impact of Propeptide Cleavage on the Stability and Activity of a Streptococcal Immunomodulatory C5a Peptidase for Biopharmaceutical Development
Source: Mol Pharm. 2023 Jul 5;20(8):4041–9. doi: 10.1021/acs.molpharmaceut.3c00207 (PMC10410607; doi:10.1021/acs.molpharmaceut.3c00207)
Supplement: Supplementary file 1 — mp3c00207_si_001.pdf [file mp3c00207_si_001.pdf]

Impact of Propeptide Cleavage on the Stability and Activity of a Streptococcal Immunomodulatory C5a Peptidase for Biopharmaceutical Development

Vinayakumar Gedi[1], Francisco Duarte[1], Pratikkumar Patel[1], Promita Bhattacharjee[1] Malgorzata Tecza[1], Kieran McGourty[1,2], Sarah P. Hudson[1,2*]

[1]Department of Chemical Sciences, Bernal Institute, University of Limerick, Limerick, V94 T9PX, Ireland

[2]SSPC SFI Research Centre for Pharmaceuticals, University of Limerick, Limerick, V94 T9PX, Ireland

[*]Email: sarah.hudson@ul.ie; Phone: +353(0)876346006

Supporting Information

Supplementary Figures S1 – S8

Supplementary Table S1

Supplementary Figures

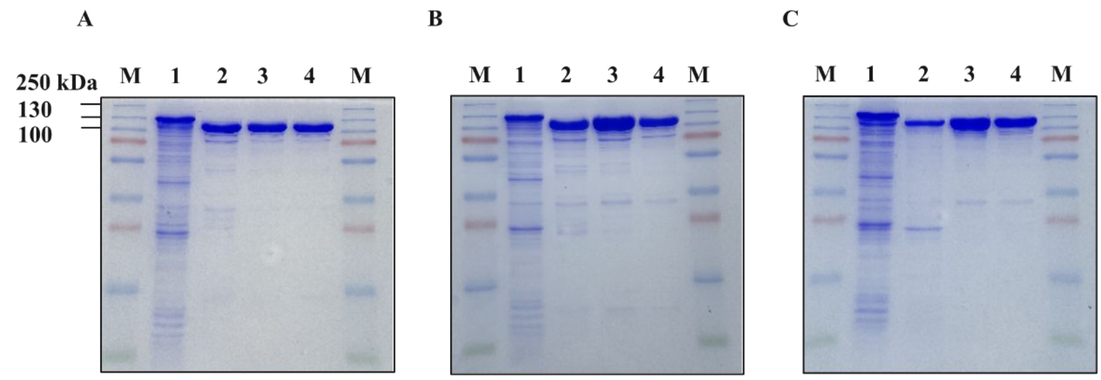

Supp Fig. S1. SDS-PAGE analysis of purification stages of ScpA variants. (A) 79ΔPro, (B) 92ΔPro and (C) ScpA. Lanes (M) Molecular weight ladder, (1) Crude cell lysate, (2) GST-affinity purified, (3) Anion-exchange purified and (4) Size-exclusion purified protein.

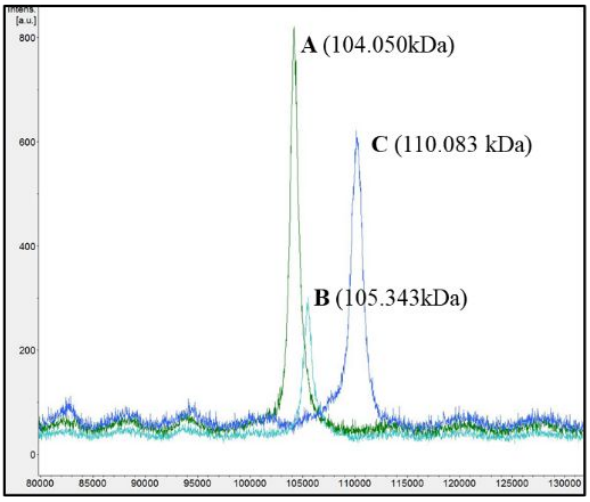

Supp Fig. S2. Intact mass spectra of purified ScpA variants. (A) 92ΔPro, (B) 79ΔPro and (C) ScpA.

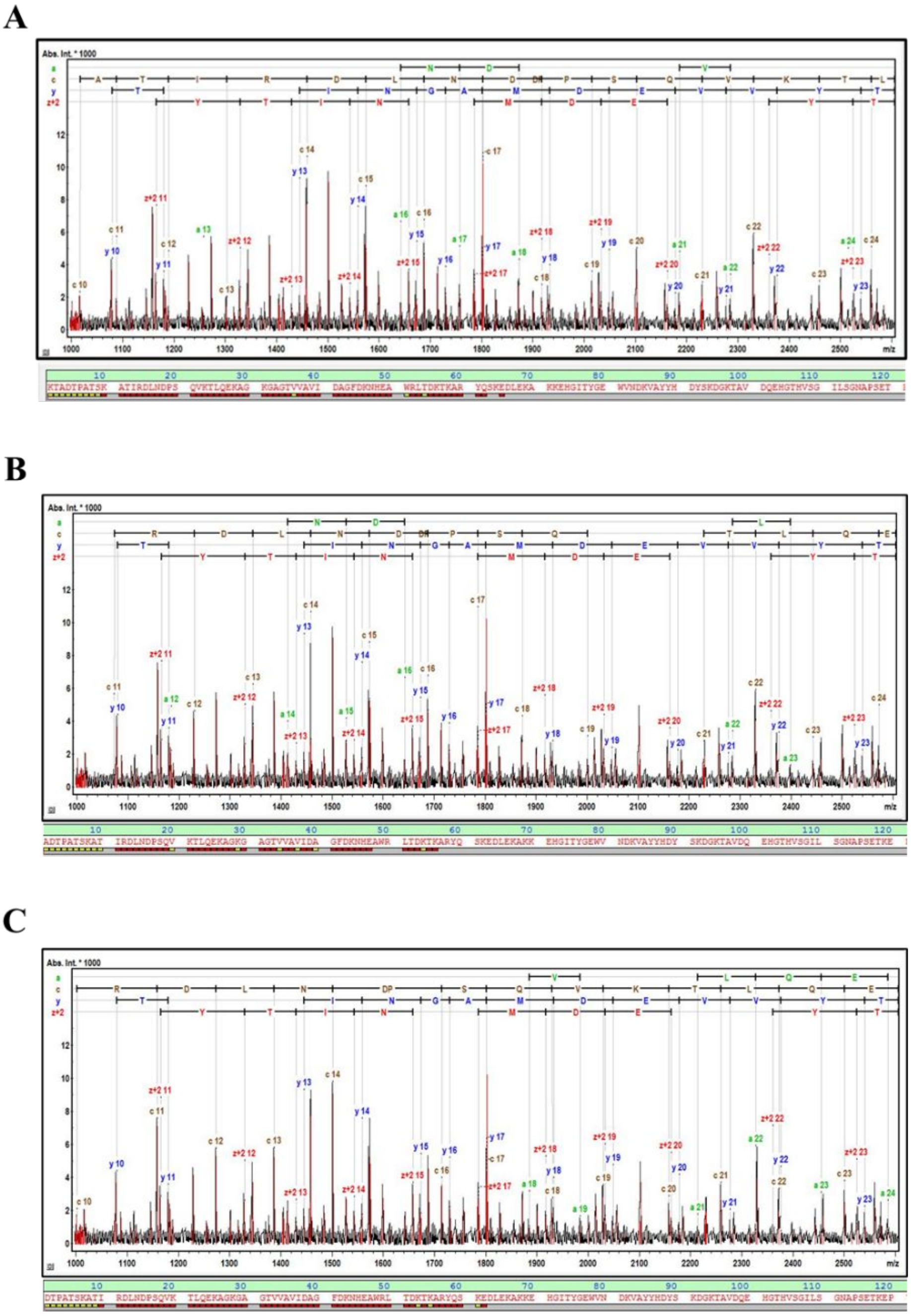

Supp Fig. S3. MALDI-ISD spectrum of mature-ScpA with matching sequence starting at K90 (A), A92 (B) and D93 (C). c and a series ions correspond to the fragments formed from fragmentation of the N-terminus, y and z^{+2} ions correspond to the fragments formed from fragmentation of the C-terminus. Red blocks in the sequence represent matched amino acids and yellow block represent predicted amino acids.

A

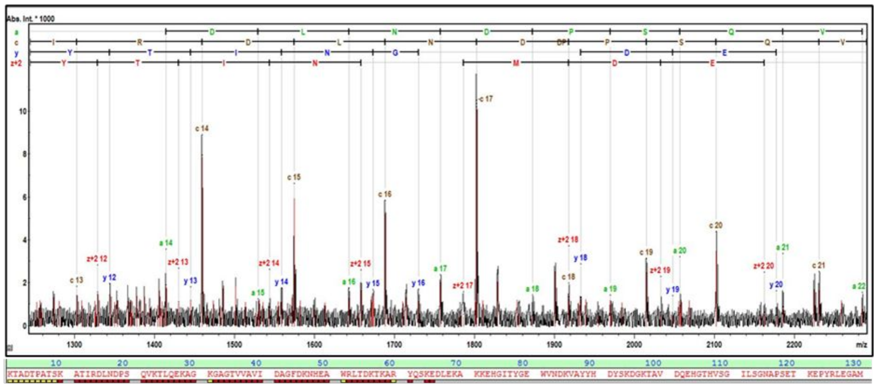

B

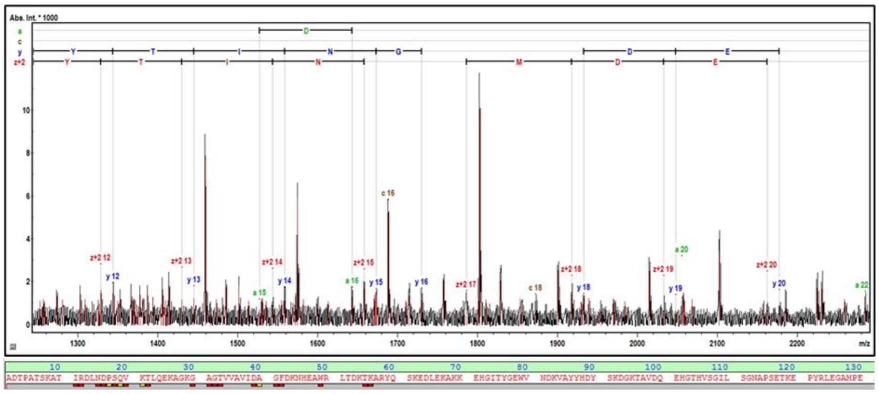

C

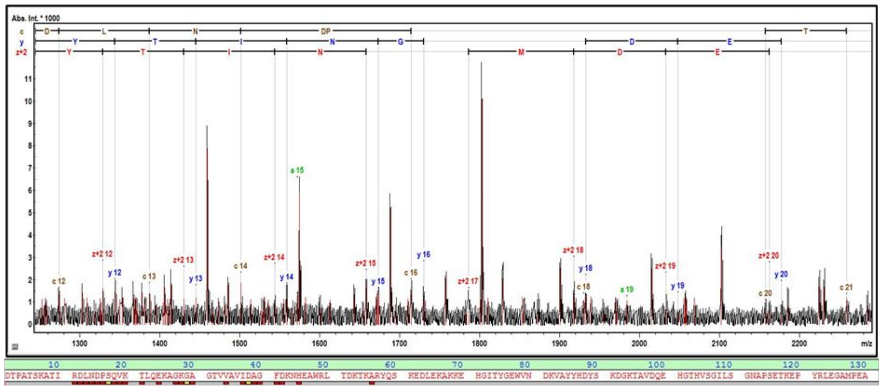

Supp Fig. S4. MALDI-ISD spectrum of ScpA with matching sequence starting at K90 (A), A92 (B) and D93 (C). No intact ScpA sequence was detected. c and a series ions correspond to the fragments formed from fragmentation of the N-terminus, y and z^{+2} ions correspond to the fragments formed from fragmentation of the C-terminus. Red blocks in the sequence represent matched amino acids and yellow block represent predicted amino acids.

A

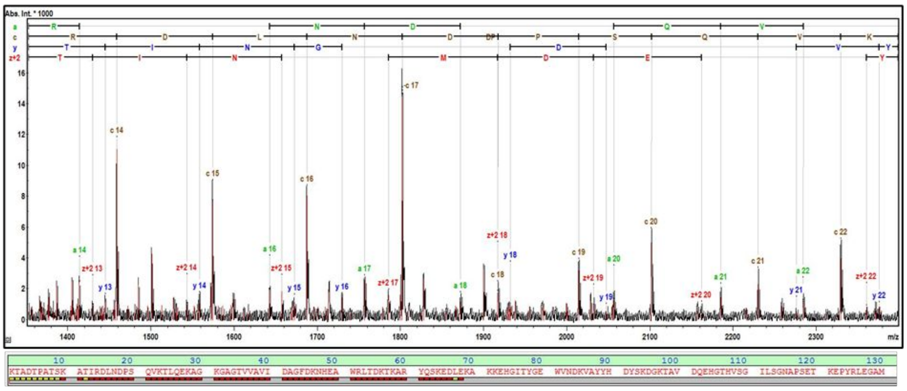

B

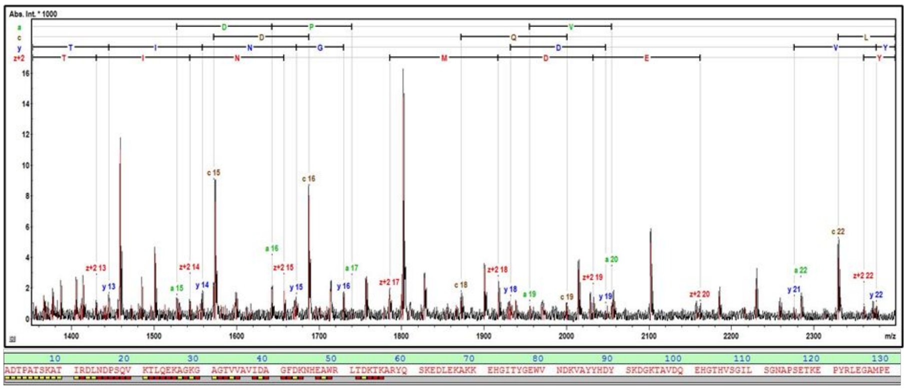

C

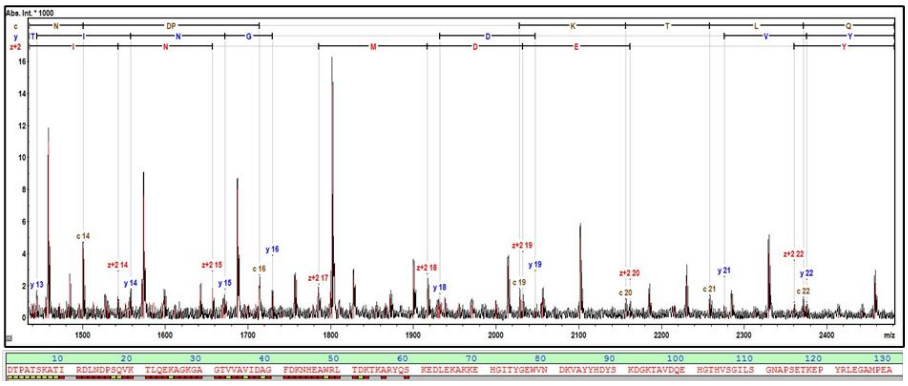

Supp Fig. S5. MALDI-ISD spectrum of 79ΔPro with matching sequence starting at K90 (A), A92 (B) and D93 (C). No intact 79ΔPro sequence was detected. c and a series ions correspond to the fragments formed from fragmentation of the N-terminus, y and z^{+2} ions correspond to the fragments formed from fragmentation of the C-terminus. Red blocks in the sequence represent matched amino acids and yellow block represent predicted amino acids.

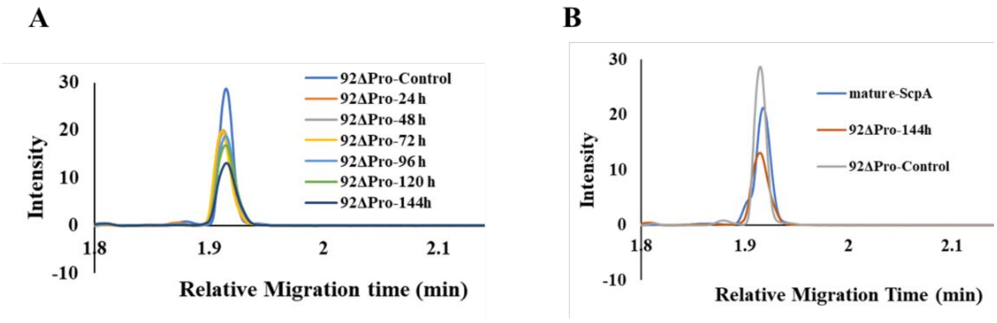

Supp Fig. S6. CE-SDS analysis of 92ΔPro. CE-SDS spectrum of (A) 92ΔPro incubated at 37 ^{o}C for 144 h and (B) mature-ScpA with 92ΔPro control (from freezer) and after incubation at 37 ^{o}C for 144 h.

A

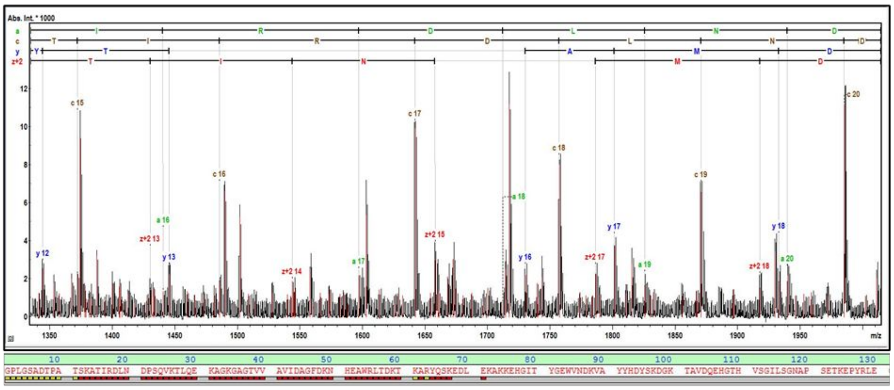

B

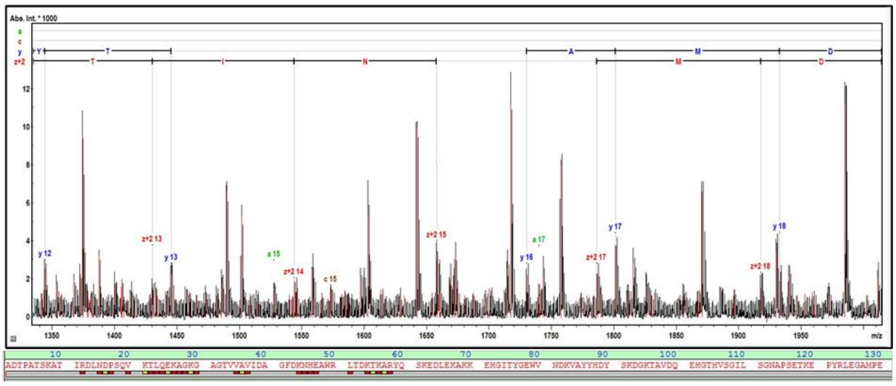

C

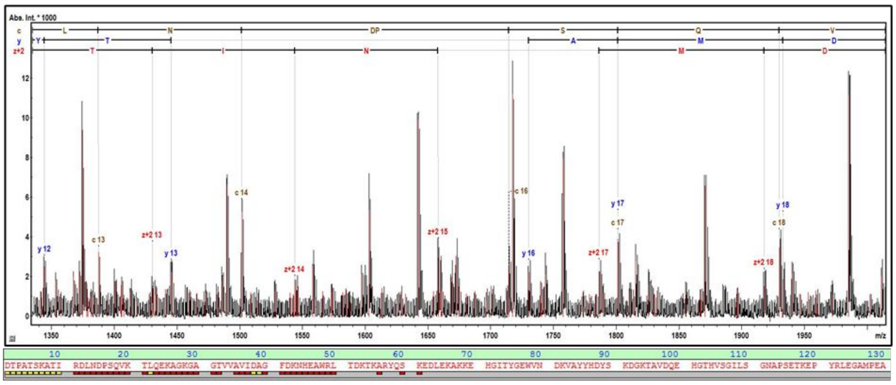

Supp Fig. S7. MALDI-ISD spectrum of 92ΔPro with matching sequence starting at Intact (A), A92 (B) and D93 (C). 'GPLGS' amino acids from intact sequence are from PGEX-plasmid used for expression and purification. c and a series ions correspond to the fragments formed from fragmentation of the N-terminus, y and z^{+2} ions correspond to the fragments formed from fragmentation of the C-terminus. Red blocks in the sequence represent matched amino acids and yellow block represent predicted amino acids.

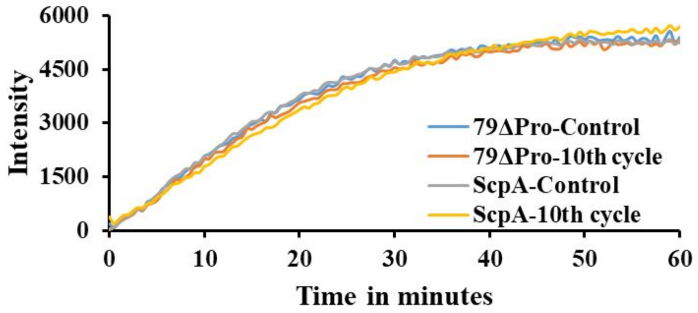

Supp Fig. S8. Activity of control and freeze-thaw subjected 79ΔPro and ScpA variants (500 pM) tested against BODIPY-FL-C75 (200 nM) in PBS-T. Initial thawed protein aliquots from -70oC freezer used as controls and their activity compared after 10 cycles of repeated freeze-thawing.

Supplementary Tables

Supp. Table 1. SPR kinetic parameters of both S512A-79ΔPro and S512A-ScpA variants against C5a

	$k_a \times 10^5$ (M^{-1} s^{-1})	$k_d \times 10^{-3}$ (s^{-1})	R_{max} (RU)	K_D (nM)
S512A-79ΔPro	1.87	4.67	75.1	35
S512A-ScpA	1.82	6.38	56.3	25
